# Clinical and cost outcomes following genomics‐informed treatment for advanced cancers

**DOI:** 10.1002/cam4.4076

**Published:** 2021-06-21

**Authors:** Deirdre Weymann, Samantha Pollard, Brandon Chan, Emma Titmuss, Alexandra Bohm, Janessa Laskin, Steven J. M. Jones, Erin Pleasance, Jessica Nelson, Alexandra Fok, Howard Lim, Aly Karsan, Daniel J. Renouf, Kasmintan A. Schrader, Sophie Sun, Stephen Yip, David F. Schaeffer, Marco A. Marra, Dean A. Regier

**Affiliations:** ^1^ Cancer Control Research BC Cancer Vancouver Canada; ^2^ School of Population and Public Health University of British Columbia Vancouver Canada; ^3^ Canada's Michael Smith Genome Sciences Centre BC Cancer Vancouver Canada; ^4^ Division of Medical Oncology BC Cancer Vancouver Canada; ^5^ Department of Medicine Faculty of Medicine University of British Columbia Vancouver Canada; ^6^ Department of Medical Genetics Faculty of Medicine University of British Columbia Vancouver Canada; ^7^ Department of Pathology & Laboratory Medicine Faculty of Medicine University of British Columbia Vancouver Canada; ^8^ Department of Molecular Oncology BC Cancer Vancouver Canada; ^9^ Hereditary Cancer Program BC Cancer Vancouver Canada; ^10^ Department of Pathology BC Cancer Vancouver Canada; ^11^ Division of Anatomical Pathology Vancouver General Hospital University of British Columbia Vancouver Canada

**Keywords:** biostatistics, genomic sequencing, healthcare costs, precision oncology, quasi‐experimental methods, treatment outcomes

## Abstract

**Background:**

Single‐arm trials are common in precision oncology. Owing to the lack of randomized counterfactual, resultant data are not amenable to comparative outcomes analyses. Difference‐in‐difference (DID) methods present an opportunity to generate causal estimates of time‐varying treatment outcomes. Using DID, our study estimates within‐cohort effects of genomics‐informed treatment versus standard care on clinical and cost outcomes.

**Methods:**

We focus on adults with advanced cancers enrolled in the single‐arm BC Cancer Personalized OncoGenomics program between 2012 and 2017. All individuals had a minimum of 1‐year follow up. Logistic regression explored baseline differences across patients who received a genomics‐informed treatment versus a standard care treatment after genomic sequencing. DID estimated the incremental effects of genomics‐informed treatment on time to treatment discontinuation (TTD), time to next treatment (TTNT), and costs. TTD and TTNT correlate with improved response and survival.

**Results:**

Our study cohort included 346 patients, of whom 140 (40%) received genomics‐informed treatment after sequencing and 206 (60%) received standard care treatment. No significant differences in baseline characteristics were detected across treatment groups. DID estimated that the incremental effect of genomics‐informed versus standard care treatment was 102 days (95% CI: 35, 167) on TTD, 91 days (95% CI: −9, 175) on TTNT, and CAD$91,098 (95% CI: $46,848, $176,598) on costs. Effects were most pronounced in gastrointestinal cancer patients.

**Conclusions:**

Genomics‐informed treatment had a statistically significant effect on TTD compared to standard care treatment, but at increased treatment costs. Within‐cohort evidence generated through this single‐arm study informs the early‐stage comparative effectiveness of precision oncology.

## Graphical abstract

Difference‐in‐difference analysis is used to address confounding when analyzing real‐world data from a single‐arm precision oncology trial. Enrolled patients receiving genomics‐informed treatment are treated longer, suggesting improved efficacy, but at an increased cost compared to those receiving standard care.

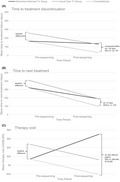

## 1 INTRODUCTION

Precision oncology aligns interventions to individual molecular alterations and candidate pathways through rapidly characterizing interpatient genomic heterogeneity. Despite potential for individual health benefit, comprehensive genomic sequencing is infrequently reimbursed by healthcare systems.^1^ Limited adoption reflects a lack of evidence on the comparative benefits of comprehensive sequencing in a tumor‐agnostic setting.^2^

Few randomized controlled trials (RCTs) have examined precision oncology’s efficacy.^3^ SHIVA is the only completed RCT to evaluate comparative effects of using a tumor‐agnostic multi‐gene panel to inform targeted therapy versus conventionally selecting therapy.^4^ SHIVA failed to detect significant health benefits, partly reflecting insufficient sample sizes for subgroup analyses, a persistent challenge when assigning therapies according to genomic‐level differences that make each cancer individually rare.^5^ Recognizing this as a barrier for RCTs, precision oncology frequently pursues single‐arm trials.^6^

To identify a comparator for single‐arm trials that helps establish causality of effect and addresses selection bias, researchers are applying matching methods.^7^, ^8^ Matching requires population‐based data that may be unavailable or incomplete in some jurisdictions. Difference‐in‐difference (DID) analysis solely requires within‐cohort data. By evaluating pre‐ and post‐sequencing outcomes, DID is able to estimate net incremental effects adjusted for baseline differences across groups, as well as aggregate factors causing changes over time. Resulting effect estimates are causal, provided that patient outcomes have followed parallel trends across groups over time.^9^

In this study, we use DID for evaluating single‐arm precision oncology data.^10^ We estimate the within‐cohort effects of genomics‐informed treatment versus standard care among patients enrolled in British Columbia’s (BC) single‐arm Personalized OncoGenomics (POG) program.^10^, ^11^ Primary outcomes include time to treatment discontinuation (TTD), time to next treatment (TTNT), and treatment costs. TTD and TTNT are intermediate clinical endpoints strongly correlated with patient response and survival outcomes.^12^, ^13^, ^14^ Evaluations of these measures are common in advanced cancers, where treatment often continues beyond objective disease progression.^15^, ^16^, ^17^ Our null hypothesis is that there are no differences in TTD, TTNT, or treatment costs across patients receiving genomics‐informed versus standard care. Our alternative hypothesis is that patients are treated with genomics‐informed therapies longer than standard care therapies, reflected by longer TTD and TTNT, and that these therapies are more expensive than standard care.

## 2 METHODS

Our retrospective cohort study uses data previously gathered in the BC Cancer POG program (NCT02155621), a single‐group research study in British Columbia, Canada.^10^, ^11^ POG uses whole‐genome and transcriptome analysis (WGTA) to characterize and interpret genomic landscapes in an interdisciplinary setting. WGTA information was considered clinically actionable if a target or risk factor was found with the potential to affect the patient’s treatment plan or if the results yielded additional information beyond what was already known from prior genetic tests.^10^ A multidisciplinary tumor board reviewed WGTA results and prioritized clinically actionable alterations and corresponding treatment options. Patients who received at least one of the treatments listed as possible WGTA‐informed actions by the tumor board were considered to be on a genomics‐informed treatment plan. Standard of care at BC Cancer instead involves using a combination of single‐gene tests, multi‐gene panels, and clinical practice guidelines to select treatment protocols for patients, including cytotoxic chemotherapies and targeted therapies that are either publicly reimbursed or accessed through BC Cancer’s Compassionate Access Program. Owing to non‐randomized enrollment and variable treatment assignment, effects of WGTA‐informed treatment on clinical outcomes and healthcare costs are poorly established.

### 2.1 Study population

Our study cohort was identified from adults who provided informed consent, enrolled in POG between July 2012 and August 2017, and received WGTA, as previously described.^11^ Eligible patients underwent biopsy, provided samples with sufficient tumor content to support reliable sequencing, and had high‐quality sequence data generated. At enrollment, POG inclusion criteria were as follows: (i) locally advanced or metastatic disease considered incurable by the oncologist; and (ii) good performance status. Participants who progressed to advanced‐stage disease years after their initial cancer diagnosis were eligible to participate in POG. Follow‐up data were available until the end of 2018, with all included individuals having a minimum of 1‐year follow up after WGTA.

**FIGURE 1:**
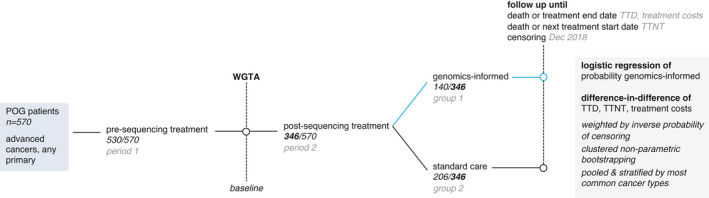
Study flow diagram

The study design and methodology are presented in Figure 1. DID requires repeated outcomes data measured across treatment groups for a minimum of two study periods. We, therefore, divided our observed time horizon, from initial cancer diagnosis until 2018, into two study periods and measured outcomes for systemic therapy treatments initiated prior to sequencing (period 1) and post‐sequencing (period 2). In period 1, all patients received a standard care treatment. After discontinuing this treatment, patients then began a new treatment in period 2. Treatment groups comprised: (1) patients whose first treatment in period 2 was classified as genomics informed by POG’s interdisciplinary tumor board; and (2) patients who instead received a standard care treatment not guided by their WGTA results in period 2.

We excluded patients who did not initiate a new line of systemic therapy in periods 1 (*n* = 40, 7%) and 2 (*n* = 184, 32%), as corresponding data were note amenable to DID. Excluded patients may have been treated with radiation therapy or surgical resection rather than systemic therapy prior to or following sequencing, for which TTD and TTNT are not evaluable. Following WGTA results, excluded patients were either pending further treatment or experienced death or disease progression before WGTA data were acted on and, thus, did not have repeated outcomes data available for DID.

### 2.2 Data sources and derived variables

BC Cancer’s linked administrative datasets captured POG enrollment, demographics, clinical characteristics, all treatment history, including therapies administered off‐label or in clinical trials, and real‐world endpoints, including TTD, TTNT, and treatment costs. We measured treatment costs from a real‐world cancer care system perspective. BC Cancer Pharmacy data capture costs for all approved systemic therapy drugs administered in regional cancer centers, community hospitals, or taken at home, as well as costs for drugs dispensed concurrently to prevent or manage toxicity. Costs for non‐approved systemic therapy drugs, including those administered in clinical trials, were imputed by multiplying list prices reported in Table S1 with either observed dosage or average dosage from cited data source, over the observed treatment period. Costs are reported in 2018 Canadian dollars. Sensitivity analysis considered drug prices as an additional outcome rather than costs over the treatment duration (reported in Supplementary Materials). We calculated patients’ TTD as the duration from treatment initiation to discontinuation due to progression, toxicity, patient preference, or death. TTNT measured duration from treatment initiation to start of next treatment or death.

### 2.3 Statistical analysis

Baseline differences were characterized across treatment groups using logistic regression of the probability of genomics‐informed treatment. The regression considered patient characteristics hypothesized to correlate with availability of and access to targeted treatments, including: age, sex, geographic area classification, primary cancer type, stage at diagnosis, and number of lines of prior systemic therapy. We selected our final model to maximize model goodness of fit as indicated by Akaike information criteria.^18^

DID estimated the effects of genomics‐informed care versus standard care on TTD, TTNT, and treatment costs. In stratified models, we assessed effect modification for the two most common primary cancer types observed within cohort. To account for censoring due to incomplete follow up, DID regressions were weighted by each individual’s inverse probability of censoring. Weights were based on product limit estimates of probability of censoring over the study period. Non‐parametric bootstrapping clustered by primary tumor site estimated standard errors. Reported confidence intervals are bias corrected.^19^ We conducted all analyses in Stata 15 (StataCorp). To identify statistical significance, we applied a threshold of *p* < 0.05, adjusted for multiple comparisons using a Bonferroni correction.^20^ Our study was approved by the UBC‐BC Cancer Research Ethics Board (Certificate No. H18‐00767).

## 3 RESULTS

From July 2012 to December 2017, 346 patients with advanced cancer were enrolled in the BC POG program and met our study criteria. Of these, 140 (40%) received a genomics‐informed treatment and 206 (60%) received standard care treatment after sequencing. A detailed list of all genomics‐informed treatments received during the period is provided in Table S2. WGTA‐informed treatments were accessed through clinical trials (4%), through off‐label or experimental use of drugs either restricted in funding or not publicly funded (38%), and as standard therapies (58%). Common reasons for not providing WGTA‐informed treatments included ongoing response to standard care treatment (*n* = 84, 42%), no clinically actionable findings generated through WGTA (*n* = 45, 23%), no targeted therapies available or accessible to patients (*n* = 23, 12%), and death or disease progression (*n* = 6, 3%).

Table 1 describes the study characteristics for our cohort. After adjusting for multiple comparisons, there were no significant baseline differences across groups. Most patients lived in urban areas (*n*_standard_ = 161, 78% and *n*_genomics_ = 106, 76%), and were diagnosed with breast cancers (*n*_standard_ = 75, 36%, *n*_genomics_ = 42, 30%) or gastrointestinal cancers (*n*_standard_ = 41, 20%, *n*_genomics_ = 30, 21%), most often colorectal. On average, participants were aged 55 (*SD*_standard_: 12.24 and *SD*_genomics_: 13.58), diagnosed with cancer in 2011 (*SD*_standard_: 5.81) or 2010 (*SD*_genomics_: 7.77), and received 2.6 (*SD*_standard_: 1.66) or 2.7 lines (*SD*_genomics_: 1.74) of systemic therapy prior to WGTA. Logistic regression results (Table S3) indicated that the probability of receiving WGTA‐informed treatment was not jointly influenced by included covariates (*p* = 0.52).

**TABLE 1:** Baseline study characteristics for patients with advanced cancer

Characteristics *N* = 346	No. (%)^α^		
	Genomics informed (*n* = 140)	Standard care (*n* = 206)	*p*‐value^τ^
Gender, female	86 (61.43)	149 (72.33)	0.033
Age at index, mean (*SD*)	54.69 (13.58)	54.68 (12.24)	0.997
Geographic area classification			0.082
Urban	106 (75.71)	161 (78.16)	
Rural	32 (22.86)	33 (16.02)	
Mixed	1 (0.71)	10 (4.85)	
LHA missing	1 (0.71)	2 (0.97)	
Primary cancer site			0.865
Breast	42 (30.00)	75 (36.41)	
Gastrointestinal	30 (21.43)	41 (19.90)	
Lung	14 (10.00)	20 (9.71)	
Sarcoma	16 (11.43)	15 (7.28)	
Pancreas	6 (4.29)	12 (5.83)	
Gynecological	10 (7.14)	15 (7.28)	
Other	22 (15.71)	28 (13.59)	
Year of diagnosis, mean (*SD*)	2009.91 (7.77)	2010.66 (5.81)	0.303
Stage at diagnosis			0.883
Stage I	14 (10.00)	21 (10.19)	
Stage II	14 (10.00)	27 (13.11)	
Stage III	10 (7.14)	16 (7.77)	
Stage IV	23 (16.43)	31 (15.05)	
REC, UNK, or NCR	79 (56.43)	111 (53.88)	
Number of lines prior to index date, mean (*SD*)	2.71 (1.74)	2.64 (1.66)	0.720

Abbreviations: *SD*, standard deviation; REC, UNK, or NCR, recurrent, stage unknown, or no classification recommended. Significance level: *p* < 0.007 (= 0.05/7) after Bonferroni correction.

α Frequencies and percentages reported for categorical variables, means, and standard deviations reported for continuous variables.

τ *p*‐value from Chi‐square tests for categorical variables and paired *t*‐tests for continuous variables.

POG enrollment was ongoing throughout the period and follow‐up times ranged from 1.12 years to 6.38 years. Median follow‐up was 2.83 years in genomics‐informed patients and 2.59 years in standard care patients. Incomplete follow up resulted in 6% of patients having censored TTD and treatment costs and 13% having censored TTNT in period 2. While the majority of treatment costs were reimbursed by BC’s public single‐payer system, 15% (*n* = 21) of patients receiving genomics‐informed treatment paid out of pocket.

### 3.1 Pooled analysis

**FIGURE 2:**
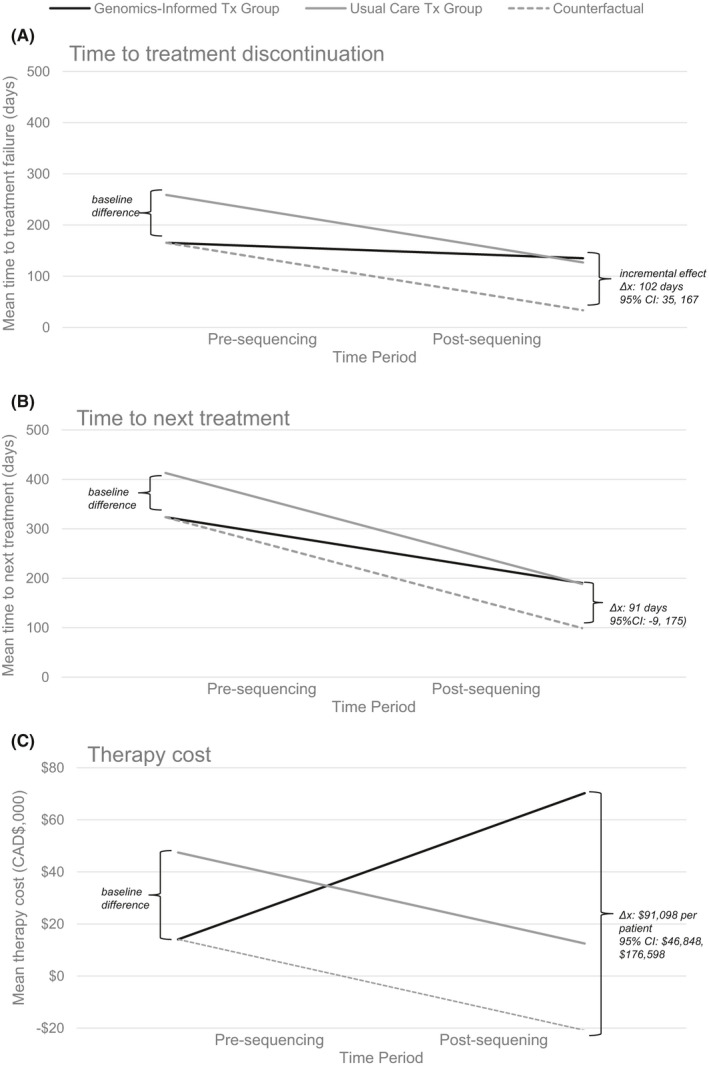
Pre‐ and post‐sequencing trends in average (A) time to treatment discontinuation; (B) time to next treatment; and (C) treatment cost

Trends in time‐varying outcomes are depicted in Figure 2. DID regression is reported in Table 2. Average TTD was significantly shorter in period 1 in genomics‐informed patients compared to standard care patients (mean difference: Δx̅_TTD,pre_ = −94 days, 95% CI: −143, −45). TTNT was shorter and treatment costs were lower but not significant (Δx̅_TTNT,pre_ = −89, 95% CI: −174, 5 and Δx̅_cost,pre_ = −$33,354, 95% CI: −$61,609, −$12,365). Pre‐sequencing clinical outcomes were, thus, poorer for patients who received WTGA‐informed treatment compared to those continuing with standard care, suggesting baseline differences in responsiveness to standard care therapies or phase of treatment.

**TABLE 2:** Difference‐in‐difference analysis of time‐varying treatment outcomes

Characteristics	Time to treatment discontinuation (days)			Time to next treatment (days)			Therapy cost (CAD$)		
	Mean	SE	*p*‐value	Mean	SE	*p*‐value	Mean	SE	*p*‐value
Pre‐sequencing									
Genomics‐informed Tx group	165.32	16.53		323.59	33.42		14,088.03	3231.48	
Standard care Tx group	258.87	19.73		412.89	31.40		47,441.95	12,634.52	
Difference pre‐sequencing (Δx̅_pre_ = x̅_genomics,pre_‐ x̅_standard,pre_)	−93.55	25.62	<0.0001	−89.31	45.03	0.047	−33,353.92	12,982.28	0.010
Post‐sequencing									
Genomics‐informed Tx group	135.18	20.52		190.30	15.67		70,227.90	28,452.51	
Standard care Tx group	126.99	11.05		188.54	13.38		12,483.97	2982.05	
Difference post‐sequencing (Δx̅_post_ = x̅_genomics,post_‐ x̅_standard,post_)	8.19	23.32	0.725	1.76	20.42	0.931	57,743.93	28,535.11	0.043
Incremental effect (Δx̅ = Δx̅_post_‐ Δx̅_pre_)	101.74	34.05	0.003	91.07	44.94	0.043	91,097.85	31,690.27	0.004

Abbreviations: SE, standard error; Tx, treatment.

Bonferroni correction was used for multiple comparisons.

^20^Significance level: *p* < 0.006 (= 0.05/9) after correction. Non‐parametric bootstrapping clustered by primary tumor site estimated standard errors. Reported confidence intervals are bias corrected. Costs are reported in 2018 Canadian dollars.

Downward time trends in average TTD and TTNT were observed irrespective of treatment group (Δx̅_TTD,standard_ = −132 days, 95% CI: −179, −93, Δx̅_TTD,genomics_ = −30 days, 95% CI: −78, 26, Δx̅_TTNT,standard_ = −224 days, 95% CI: −296, −168, and Δx̅_TTNT, genomics_ = −133 days, 95% CI: −216, −79), whereas trends in treatment costs were opposite across groups (Δx̅_cost,standard_ = −$34,958, 95% CI: −$64,344, −$13,729; Δx̅_costs,genomics_ = $56,140, 95% CI: $15,490, $136,701). Trends are indicative of increasingly shorter response times on subsequent lines of therapy as treatment resistance develops.

Accounting for baseline differences and observed time trends, DID estimated that genomics‐informed versus standard care treatment had a statistically significant incremental effect of 102 days (95% CI: 35, 167) on TTD at a significantly increased treatment cost of $91,098 per patient (95% CI: $46,848, $176,598). Sensitivity analysis reported in Table S4 revealed no significant differences in drug prices across groups, demonstrating that higher treatment costs primarily resulted from extended durations of use. Although TTNT was higher after genomics‐informed treatment, this difference was not significant (91 days, 95% CI: −9, 175), with estimates suggesting less durable response and lower efficacy for standard care.

### 3.2 Stratified analysis

Stratifying our analysis according to the two most common cancer types revealed outcomes heterogeneity that was masked in the pooled results. Table S5 presents stratified regressions for breast, gastrointestinal, and other cancers.

### 3.3 Breast cancer (n = 117)

Similar to pooled analysis, we found that period 1 treatment costs for breast cancer were significantly lower among genomics‐informed patients (Δx̅_cost,pre_ $−16,981. 95% CI:−$29,412, −$7,753) compared to usual care. We also found downward trends in TTNT over time among genomics‐informed and usual care patients (Δx̅_TTNT,standard_ = −210 days, 95% CI: −347, −122, and Δx̅_TTNT, genomics_ = −126 days, 95% CI: −218, −43) and downward trends in TTD and treatment costs among usual care patients (Δx̅_TTD,standard_ = −72 days, 95% CI: −128, −11 and Δx̅_cost,standard_ = −$16,976, 95% CI: −$29,989, −$8135).

Key differences among breast cancer patients were that no significant baseline differences in TTD or TTNT were present across patient groups and no significant trends in TTD or treatment costs were observed in genomics‐informed patients. Within breast cancers, WGTA‐informed treatment did not have a statistically significant incremental effect on TTD, TTNT, or treatment costs compared to standard care.

### 3.4 Gastrointestinal cancer (n = 71)

Stratified results for patients with gastrointestinal cancers were largely consistent with pooled results. Significantly lower baseline outcomes were present in genomics‐informed versus usual care patients (Δx̅_TTD,pre_ = −295, 95% CI: −500, −169; Δx̅_TTNT,pre_ = 312, 95% CI: −551, −194; Δx̅_cost,pre_ = −31,230, 95% CI: −$46,896, −19,659) and downward trends in TTD, TTNT, and treatment costs were detected among usual care patients (Δx̅_TTD,standard_ = −298 days, 95% CI: −512, −171; Δx̅_TTNT,standard_ = −370 days, 95% CI: −632, −279; and Δx̅_cost,standard_= −$23,375, 95% CI: −$38,301, −$7471). For gastrointestinal cancers, patients who received WGTA‐informed treatment had significantly higher TTD, TTNT, and treatment cost compared to standard care. Incremental effects ranged from 284 days (95% CI: 138, 525) on TTD to 327 days (95% CI: 202, 623) on TTNT to $53,861 (95% CI: $16,250, $126,342) on treatment costs.

### 3.5 Other cancers (n = 158)

Among patients diagnosed with other cancers, estimated magnitudes and directions often aligned with pooled results, although few differences remained significant after correcting for multiple comparisons (*p* > 0.006). In period 1, genomics‐informed patients had weakly significantly shorter TTD (Δx̅_TTD,pre_ = −77, 95% CI: −139, −24). Significant downward trends in TTD and TTNT were observed among usual care patients (Δx̅_TTD,standard_ = −106, 95% CI: −156, −61; Δx̅_TTNT,standard_ = −170, 95% CI: −245, −98). Patients with other cancers who received WGTA‐informed treatment experienced longer TTD (*p* = 0.054) and higher treatment costs (*p* = 0.021), although these were no longer statistically significant after Bonferroni correction. Incremental effects included 74 days (95% CI: 5, 153) on TTD and $88,476 (95% CI: $27,920, $183,004) on treatment costs.

## 4 DISCUSSION

Ours is the first study to apply DID for evaluating single‐arm precision oncology trials. We found that after undergoing WGTA in an advanced cancer setting, resulting data were used in 40% of patients to guide treatment, either through rationally selecting a standard care option where multiple such options exist or through identifying off‐label, clinical trial or not approved treatments where standard care options were ill‐defined, undesirable, or non‐existent. We estimated that patients received genomics‐informed treatment over 3 months longer than standard care (102 days; 95% CI: 35, 167), aligning with improved progression‐free survival ratios observed in prior within‐patient evaluations.^21^, ^22^ This relative magnitude is promising in the metastatic cancer setting, where incremental efficacy gains for reimbursed treatments are typically measured in months rather than years.^23^

Estimated effects varied across tumor types. Genomics‐informed treatment resulted in significantly longer TTD and TTNT within gastrointestinal cancers (*p* = 0.002 and *p* < 0.0001) and longer albeit not significant TTD in other cancers (*p* = 0.054), but had no effect within breast cancers. The benefits of tumor‐agnostic precision oncology will, thus, be driven by heterogeneous responses across cancer subtypes. Our results confirm that uniform coverage of genomic sequencing across all indications is unlikely to yield equal effectiveness or cost‐effectiveness for all patients.^24^ Shifting focus to phenotypes most likely to benefit from genomics‐informed treatment will balance the need to maximize population health outcomes with the recognition that sequencing introduces opportunity costs. Clinicians can use evidence generated in this study to transparently communicate outcomes heterogeneity and expected benefits to patients.

Longer TTD directly influences costs borne by healthcare systems and by patients facing out‐of‐pocket payments. Accessing genomics‐informed treatment increased mean expenditures for patients by $91,098 (95% CI: $46,848, $176,598). Genomics‐informed treatments cost more than standard care treatments, primarily owing to longer durations of use rather than high prices of on‐patent treatments. This effect was most prominent in gastrointestinal cancers. Higher treatment costs combined with upfront sequencing costs will have implications on cancer care budgets and the ability to pay for genomics‐guided interventions, whether by public healthcare systems, private payers, or patients.^25^, ^26^ Such pressures may introduce equity and access issues for patients within and across jurisdictions.

Prior to sequencing, standard care patients had significantly higher TTD and treatment costs compared to patients receiving WGTA‐informed treatment, despite having similar enrollment characteristics. While unadjusted analyses across these patient groups would result in biased effect estimates, baseline outcomes differences do not directly threaten the validity of DID estimates.^27^ Observed differences do suggest possible variation in either responsiveness to previous standard care therapies or treatment phase. Sensitivity analysis excluding patients who did not receive genomics‐informed treatment because of ongoing response to standard care (*n* = 48) did not substantively change effect estimates (Table S6). Furthermore, potential non‐constant trends in outcomes across treatment phases are most likely to underestimate true effects of genomics‐informed care owing to established patterns of acquired treatment resistance.^28^

To our knowledge, ours is the first study to estimate the comparative effectiveness of genomics‐informed versus standard care treatment and the first to consider DID for evaluating single‐arm precision oncology initiatives. The DID approach is common in health research and is well suited to within‐cohort estimation of causal time‐varying treatment effects.^29^, ^30^, ^31^, ^32^, ^33^ For determining efficacy and effectiveness, this method necessitates patient‐level outcomes data amenable to repeated measurement. Although survival is observed only once at an individual level and is not suitable for DID, time‐varying patients endpoints are commonly used to characterize early‐stage impacts of cancer therapies while long‐term evidence is being generated.^34^, ^35^ While real‐world time‐varying endpoints measured in this study correlate with common efficacy endpoints for certain advanced cancers, including treatment response, progression, and survival, their performance in a tumor‐agnostic population is not yet established.^12^, ^13^, ^14^

Owing to our study’s small sample size, subgroup analysis was only possible in select cancer subtypes. Future research powered to detect differences across all cancers is necessary to guide decision‐making. Treatment costs were also estimated using list prices where internal estimates were unavailable, which may overestimate actual expenditures. DID requires repeated outcomes data and POG patients who did not initiate a new treatment after WGTA, either owing to ongoing treatment response or death or disease progression, were not eligible for this study, affecting the cohort composition and generalizability of our study findings. Causality of our results also relies on the parallel trends assumption underlying DID analysis, which cannot be tested for in a two‐period setting. While early‐stage analyses of short‐term outcomes are least likely to be severely impacted by deviations from this assumption, there is an unmet need for quasi‐experimental methods development robust to time‐varying confounding.

## 5 CONCLUSIONS

In the continued absence of RCTs, DID is a tool to address confounding bias for precision oncology. By expanding the application of DID methods to evaluate single‐arm trial data, we find that genomics‐informed treatment has a statistically significant effect on TTD and treatment costs compared to standard care treatment in patients with advanced cancers. Our study will guide future quasi‐experimental within‐cohort evaluations enabling early‐stage estimation of precision oncology’s clinical and economic impacts. Broadening the spectrum of evidence generated throughout the technology life cycle will provide critical support for reimbursement decision‐making.

## CONFLICT OF INTEREST

Brandon Chan, Steven J.M. Jones, and Marco A. Marra report no conflicts of interest. Deirdre Weymann and Samantha Pollard codirect IMPRINT Research Consulting and have consulted for Roche Canada. Janessa Laskin has received honoraria for academic talks from: Roche Canada, Pfizer Canada, Astra‐Zeneca Canada, and BI Canada; her institution has received research funding for her projects from: Roche Canada, Asta‐Zeneca Canada, and BI Canada. Daniel J. Renouf disclosures include research funding and honoraria from Bayer and Roche, and travel funding and honoraria from Servier, Celgene, Taiho, Ipsen, and Astra Zenec. Howard Lim has received honoraria from Eisai, Taiho, Roche, Lilly, BMS, Amgen, and Leo for consultant work and is an investigator on trials with Bayer, BMS, Lilly, Roche, Astra‐Zeneca, and Amgen. Sophie Sun has received research grant and honoraria funding from Astra‐Zeneca. Stephen Yip is an advisory board member for and has received travel allowance from Amgen, AstraZeneca, Bayer, Norvatis, and Roche. Dean A. Regier has received speaking honoraria from Roche Canada. DF Schaeffer has received honoraria from Alimentiv, Pfizer, Merck, and Diaceutics. Kasmintan A. Schrader has received speaking honoraria from AstraZeneca and Pfizer.

## AUTHOR CONTRIBUTIONS

Study conception and design: All authors. Statistical analysis: DW, BC, and DAR. Drafting of the manuscript and final approval: All authors. Guarantor of work: DW and DAR.

## Supporting information

Table S1‐S6

## Data Availability

Patient‐level administrative data used in this retrospective study are confidential and are not available in a public repository, in accordance with institutional policies.
