# Supplementary material for: Clinical and cost outcomes following genomics‐informed treatment for advanced cancers
Source: Cancer Med. 2021 Jun 21;10(15):5131–40. doi: 10.1002/cam4.4076 (PMC8335838; doi:10.1002/cam4.4076)
Supplement: Supplementary file 1 — Table S1‐S6 [file CAM4-10-5131-s001.docx]

**SUPPLEMENTAL TABLES AND FIGURES**
Supplemental Table 1 – List prices used to impute treatment costs not captured by BC Cancer pharmacies

| Drug Name | List Price | Source |
| --- | --- | --- |
| Abraxane | $971.00 per 100mg vial | CADTH/pCODR |
| Accutane | $1.01 per 10mg | Pharmanet |
| Acetylsalicylic acid | $0.04 per 325g tablet | Pharmanet |
| Afatinib | $80.00 per 40mg tablet | CADTH/pCODR |
| Ags67e | $5131.09 per mg | Average cost of the following antibody-drug conjugates: Brentuximab, Trastuzumab, Gemtuzumab, and Inotuzumab as listed by CADTH/pCODR |
| Alectinib | $42.17 per 150 mg capsule | CADTH/pCODR |
| Anastrozole | $1.27 per mg | CADTH/pCODR |
| Atezolizumab | $5.65 per mg | CADTH/pCODR |
| Avelumab | $1,325.00 per 200 mg vial | CADTH/pCODR |
| Axitinib | $18.60 per mg | CADTH/pCODR |
| Azd5363 | $390.00 per 50mg | https://www.adooq.com/azd5363.html |
| Bevacizumab | $600.00 per 100mg vial | CADTH/pCODR |
| Bms-986115 | $728.00 per 50mg | https://www.adooq.com/ro4929097.html\ |
| Bms-986205 | $1,014.00 50mg | https://www.adooq.com/bms-986205.html |
| Bromocriptine | $1.61 per 5mg | Pharmanet |
| Cabozantinib | $293.33 per 60mg tablet | CADTH/pCODR |
| Candesartan | $0.31 per 8mg tablet | Pharmanet |
| Capecitabine | $1.53 per 500mg tablet | CADTH/pCODR |
| Carboplatin | $0.10 per mg | CADTH/pCODR |
| Cediranib | $260.00 per 50mg | https://www.adooq.com/cediranib-azd2171.html |
| Ceritinib | $67.47 per 150mg tablet | CADTH/pCODR |
| Cisplatin | $2.70 per mg | CADTH/pCODR |
| Chlordiazepoxide | $0.12 per 5mg capsule | Pharmanet |
| Cobimetinib | $120.00 per 20mg tablet | CADTH/pCODR |
| Crizotinib | $0.52 per mg | CADTH/pCODR |
| Cx-5461 | $585.00 per 50mg | https://www.adooq.com/cx-5461.html |
| Dabrafenib | $42.22 per 50mg capsule | CADTH/pCODR |
| Demcizumab | $682.85 per mg | Average cost of the following monoclonal antibodies: Alemtuzumab, Bevacizumab, Cetuximab, Gemtuzumab, Ipilimumab, Ofatumumab, Panitumumab, and Pembrolizumab as reported by CADTH/pCODR |
| Dexamethasone | $1,400.00 per implant | CADTH/pCODR |
| Dexrazoxane | $663.37 per 250 mg | https://www.drugs.com/price-guide/zinecard |
| Doxorubicin | $5.05 per mg | CADTH/pCODR |
| Durvalumab | $3911.11 per 500mg | CADTH/pCODR |
| Enzalutamide | $29.19 per 40mg capsule | CADTH/pCODR |
| Eribulin | $540.00 per mg | CADTH/pCODR |
| Etoposide | $0.75 per mg | CADTH/pCODR |
| Everolimus | $69,927.00 annually | CADTH/pCODR |
| Filgrastim | $144.31 per syringe | CADTH/pCODR |
| Fluorouracil | $0.003 per mg | CADTH/pCODR |
| Folfox | $10.20 per mg | CADTH/pCODR |
| Fulvestrant | $582.90 per 250mg/5ml injection | CADTH/pCODR |
| Gemcitabine | $6.00 per 200mg vial | CADTH/pCODR |
| Glesatinib (Mgcd265) | $845.00 per 50mg | https://www.adooq.com/mgcd-265.html |
| Herceptin | $2,700.00 per 440mg vial | CADTH/pCODR |
| Imgn853 | $5,131.09 per mg | Average cost of the following antibody-drug conjugates: Brentuximab, Trastuzumab, Gemtuzumab, and Inotuzumab as listed by CADTH/pCODR |
| Iodine-131 | €379.00 per vial | https://www.ncbi.nlm.nih.gov/pubmed/26240230 |
| Ipilimumab | $5,800.00 per 50mg vial | CADTH/pCODR |
| Irbesartan | $1.37 per 175mg tablet | Pharmanet |
| Lcl161 | $234.00 per 25mg | https://www.adooq.com/lcl-161.html |
| Lenvatinib | $8.14 per mg | CADTH/pCODR |
| Liposomal | $341.50 per 10mg vial | CADTH/pCODR |
| Lutetium-177 | $35,000.00 per dose | CADTH/pCODR |
| Ly3076226 | $5,131.09 per mg | Average cost of the following antibody-drug conjugates: Brentuximab, Trastuzumab, Gemtuzumab, and Inotuzumab as listed by CADTH/pCODR |
| Mek162 | $117.00 per 50mg | https://www.adooq.com/mek162-arry-438162.html |
| Metformin | $0.05 per 500mg tablet | Pharmanet |
| Monalizumab (Iph2201) | $682.8548823 per mg | Average cost of the following monoclonal antibodies: Alemtuzumab, Bevacizumab, Cetuximab, Gemtuzumab, Ipilimumab, Ofatumumab, Panitumumab, and Pembrolizumab as stated by CADTH/pCODR |
| Naringenin | $260.00 per 250mg | https://www.adooq.com/naringenin.html |
| Nivolumab | $782.22 per 40mg vial | CADTH/pCODR |
| Olaparib | $16.75 per 50mg capsule | CADTH/pCODR |
| Olaratumab | $788.12 per 190mg vial | CADTH/pCODR |
| Osimertinib | $294.68 per 80mg tablet | CADTH/pCODR |
| Paclitaxel | $2.00 per mg | CADTH/pCODR |
| Paclitaxel-nab | $971.00 per 100mg vial | CADTH/pCODR |
| Palbociclib | $253.90 per 125mg | CADTH/pCODR |
| Pazopanib | $41 per 200mg tablet | CADTH/pCODR |
| Pegfilgrastim | $2504.97 per 6mg | CADTH/pCODR |
| Pembrolizumab | $2,200.00 per 50mg vial | CADTH/pCODR |
| Ramucirumab | $909.42 per 100mg vial | CADTH/pCODR |
| Regorafenib | $72.62 per 40mg tablet | CADTH/pCODR |
| Reolysin | $65,000.00 USD per treatment | Cost of oncolytic virus treatment Talimogene laherparepve (T-VEC) used as an estimate (CADTH/pCODR) |
| Ribociclib | $99.20 per 200mg tablet | CADTH/pCODR |
| Rociletinib (Co-1686) | $208.00 per 50mg | https://www.adooq.com/co-1686.html |
| Romidepsin | $2,582.00 per 10mg vial | CADTH/pCODR |
| Rucaparib | $122.52 (USD) per 300mg | https://www.ncbi.nlm.nih.gov/pmc/articles/PMC6386009/#!po=37.8049 |
| Sorafenib | $46.47 per 200mg tablet | CADTH/pCODR |
| Sunitinib | $126.30 per 25mg capsule | CADTH/pCODR |
| Taselisib | $377.00 per 50mg | https://www.adooq.com/gdc-0032.html |
| Temozolomide | $1.43 per 20mg capsule | https://acsjournals.onlinelibrary.wiley.com/doi/full/10.1002/cncr.23297 |
| Temsirolimus | $1,112.55632 per 25mg | https://onlinelibrary.wiley.com/doi/full/10.1111/j.1524-4733.2009.00617.x |
| Trabectedin | $3,061.33 per mg | CADTH/pCODR |
| Trametinib | $290.00 per 2mg | CADTH/pCODR |
| Tremelimumab | $48.96 per mg | Average cost of the following immune checkpoint blockers: Nivolumab, Pembrolizumab, Ipililumab, Atezolizumab, Cemiplimab, Avelumab, and Durvalumab as listed by CADTH/pCODR |
| Vandetanib | $97.50 per 100mg tablet | CADTH/pCODR |
| Vemurafenib | $46.54 per 240mg tablet | CADTH/pCODR |
| Vorinostat | $173.37 per 100mg capsule | https://www.drugs.com/price-guide/zolinza |

Supplemental Table 2 – List of WGTA-informed treatment regimens dispensed during period

| Drug name |
| --- |
| Accutane |
| Afatinib |
| Anastrozole, Enzalutamide |
| Asa |
| Atezolizumab/Placebo, Paclitaxel-nab, MPDL3280A/Placebo |
| Bevacizumab |
| Bicalutamide |
| BMS-986115 |
| Bromocriptine, Chlordiazepoxide HCl |
| Cabozantinib |
| Candesartan |
| Capecitabine |
| Capecitabine, Oxaliplatin |
| Carboplatin, Irinotecan |
| Cetuximab |
| Cetuximab, Dabrafenib |
| Cetuximab, Sorafenib |
| CO-1686 |
| Cobimetinib |
| Cobimetinib, Vemurafenib |
| Crizotinib |
| CX-5461 |
| Enzalutamide |
| Erlotinib |
| Etoposide |
| Everolimus |
| Everolimus, Erlotinib |
| Everolimus, Exemestane |
| Gemcitabine, Cisplatin |
| Goserelin, Ribociclib, Letrozole |
| Herceptin |
| Imatinib |
| IPH2201 |
| Ipilimumab |
| Irbesartan |
| Irinotecan |
| Irinotecan, Fluorouracil |
| Irinotecan, Fluorouracil, Bevacizumab |
| Letrozole, Goserelin |
| LY3076226 |
| Mesna, Ifosfamide |
| Mesna, Ifosfamide, Etoposide |
| MGCD265 |
| MPDL3280A |
| Naringenin |
| Nilotinib |
| Nivolumab |
| Nivolumab, BMS-986205 |
| Octreotide |
| Olaparib |
| Olaparib, Cediranib |
| Osimertinib |
| Oxaliplatin |
| Paclitaxel |
| Paclitaxel, Carboplatin |
| Palbociclib |
| Palbociclib, Letrozole |
| Panitumumab |
| Pazopanib |
| Pembrolizumab |
| Pertuzumab, Trastuzumab, Vinorelbine |
| Regorafenib |
| Sorafenib |
| Sunitinib |
| Taselisib/Placebo, Fulvestrant |
| Temsirolimus |
| Trametinib |
| Trametinib, Ribociclib, Trametinb |
| Trastuzumab Emtansine |
| Trastuzumab, Paclitaxel-nab, Paclitaxel, Carboplatin, |
| Trastuzumab, Pertuzumab, Docetaxel |
| Tremelimumab, Durvalumab |
| Tremelimumab, Pemetrexed, Durvalumab, Carboplatin |
| Vorinostat |

WGTA-informed treatments listed above were highly individualized. Selection of these treatments was based on a combination of genomic alterations as well as candidate pathways dysregulated in patient’s tumors.

Supplemental Table 3– Logistic regression of probability of genomics-informed treatment

| Covariate | OR | SE | p-value |
| --- | --- | --- | --- |
| Intercept | 0.29 | 0.23 | 0.122 |
| Sex, female | 1.54 | 0.46 | 0.151 |
| Age at index | 1.00 | 0.01 | 0.622 |
| Rurality |  |  |  |
| Urban | Ref. |  |  |
| Rural | 0.18 | 0.20 | 0.113 |
| Mixed | 1.41 | 0.41 | 0.237 |
| LHA missing | 0.80 | 1.00 | 0.855 |
| Primary cancer site |  |  |  |
| Breast | Ref. |  |  |
| Gastrointestinal (excl. pancreas) | 1.31 | 0.59 | 0.550 |
| Lung | 1.40 | 0.77 | 0.538 |
| Sarcoma | 1.86 | 0.98 | 0.236 |
| Pancreas | 0.84 | 0.55 | 0.794 |
| Gynecology | 1.41 | 0.76 | 0.531 |
| Other | 1.31 | 0.65 | 0.582 |
| Stage at diagnosis |  |  |  |
| Stage I | Ref. |  |  |
| Stage II | 0.78 | 0.38 | 0.607 |
| Stage III | 0.91 | 0.52 | 0.875 |
| Stage IV | 1.14 | 0.58 | 0.798 |
| REC, UNK, NCR | 0.92 | 0.44 | 0.865 |
| Number of lines prior to index date | 1.13 | 0.10 | 0.151 |
| Pseudo R^2^ | 0.03 | | |
| Log-likelihood | -225.97 | | |
| Sample Size | 346 | | |
| Likelihood-ratio χ^2^ test statistic | 15.05 | | |
| p-value | 0.521 | | |

OR: adjusted odds ratio; SE: standard error; WGTA: whole-genome and transcriptome analysis; REC, UNK, NCR: Recurrent, unknown stage, or no classification recommended. Significance level: p<0.003 (=0.05/17) after Bonferroni correction.

Supplemental Table 4 – Difference-in-difference analysis of drug prices

| Characteristics | Sample statistic, mean (SE) | | |
| --- | --- | --- | --- |
|  | Drug Price Per Day (CAD$) | | |
|  | Mean | SE | p-value |
| Pre-sequencing |  |  |  |
| Genomics-informed Tx Group | 315.86 | 164.42 |  |
| Usual Care Tx Group | 596.53 | 275.88 |  |
| Difference pre-sequencing  (Δx̅_pre_= x̅_Genomics-informed,pre_- x̅_Usual care,pre_) | -280.67 | 320.40 | 0.381 |
| Post-Sequencing |  |  |  |
| Genomics-informed Tx Group | 6,532.56 | 5,155.24 |  |
| Usual Care Tx Group | 185.47 | 41.70 |  |
| Difference post-sequencing  (Δx̅_post_= x̅_Genomics-informed,post_- x̅_Usual care,post_) | 6,347.09 | 5,157.01 | 0.218 |
| Incremental Effect (Δx̅=Δx̅_post_- Δx̅_pre_) | 6,627.76 | 5,187.04 | 0.201 |

SE: standard error; WGTA: whole-genome and transcriptome analysis; Prices are estimated per day and reported in 2018 Canadian dollars. Significance level for within cancer type analyses: p<0.006 (=0.05/9) after Bonferroni correction.

Supplemental Table 5 – Difference-in-difference analysis of time-varying outcomes stratified by cancer type

| Characteristics | Sample statistic, mean (SE) | | | | | | | | |
| --- | --- | --- | --- | --- | --- | --- | --- | --- | --- |
|  | Time to Treatment Discontinuation (days) | | | Time to Next Treatment (days) | | | Therapy Cost  (CAD$) | | |
|  | Mean | SE | p-value | Mean | SE | p-value | Mean | SE | p-value |
| Breast Cancer (n=117) | | | | | | | | | |
| Pre-sequencing |  |  |  |  |  |  |  |  |  |
| Genomics-informed Tx Group | 218.50 | 43.57 |  | 312.36 | 47.97 |  | 5,452.75 | 1,902.26 |  |
| Usual Care Tx Group | 209.12 | 23.11 |  | 354.92 | 54.02 |  | 22,433.30 | 5,147.50 |  |
| Difference pre-sequencing  (Δx̅_pre_= x̅_Genomics-informed,pre_- x̅_Usual care,pre_) | 9.38 | 48.00 | 0.845 | -42.56 | 72.31 | 0.556 | -16,980.55 | 5,389.94 | 0.002 |
| Post-Sequencing |  |  |  |  |  |  |  |  |  |
| Genomics-informed Tx Group | 179.13 | 53.00 |  | 185.95 | 29.10 |  | 116,965.21 | 84,301.17 |  |
| Usual Care Tx Group | 136.83 | 23.17 |  | 145.35 | 20.23 |  | 5,456.91 | 1,652.28 |  |
| Difference post-sequencing  (Δx̅_post_= x̅_Genomics-informed,post_- x̅_Usual care,post_) | 42.29 | 57.77 | 0.464 | 40.60 | 35.18 | 0.248 | 111,508 | 84,308.73 | 0.186 |
| Incremental Effect (Δx̅=Δx̅_post_- Δx̅_pre_) | 32.91 | 69.47 | 0.636 | 83.17 | 70.72 | 0.240 | 128,488.85 | 84,607.55 | 0.129 |
| Gastro-Intestinal Cancer (excl. Pancreas) (n=71) | | | | | | | | | |
| Pre-sequencing |  |  |  |  |  |  |  |  |  |
| Genomics-informed Tx Group | 134.00 | 22.38 |  | 270.10 | 39.15 |  | 7,508.24 | 1,709.70 |  |
| Usual Care Tx Group | 428.83 | 78.54 |  | 582.41 | 91.76 |  | 38,738.45 | 6,362.41 |  |
| Difference pre-sequencing (Δx̅_pre_) | -294.83 | 81.22 | <0.0001 | -312.31 | 99.49 | 0.002 | -31,230.20 | 6,654.57 | <0.0001 |
| Post-Sequencing |  |  |  |  |  |  |  |  |  |
| Genomics-informed Tx Group | 120.12 | 34.21 |  | 226.66 | 48.47 |  | 37,994.74 | 25,768.86 |  |
| Usual Care Tx Group | 131.22 | 24.54 |  | 212.44 | 29.99 |  | 15,363.50 | 4,827.58 |  |
| Difference post-sequencing (Δx̅_post_) | -11.10 | 42.02 | 0.792 | 14.21 | 57.55 | 0.805 | 22,631.24 | 26,163.89 | 0.387 |
| Incremental Effect (Δx̅) | 283.73 | 93.15 | 0.002 | 326.53 | 93.15 | <0.0001 | 53,861.45 | 27,171.15 | 0.047 |
| Other Cancers (n=158) | | | | | | | | | |
| Pre-sequencing |  |  |  |  |  |  |  |  |  |
| Genomics-informed Tx Group | 146.29 | 17.84 |  | 354.12 | 61.79 |  | 22,324.43 | 6,471.73 |  |
| Usual Care Tx Group | 222.90 | 23.41 |  | 383.98 | 37.44 |  | 72,247.43 | 28,580.52 |  |
| Difference pre-sequencing (Δx̅_pre_) | -76.61 | 29.52 | 0.009 | -29.86 | 72.56 | 0.681 | -49,923.00 | 29,230.74 | 0.088 |
| Post-Sequencing |  |  |  |  |  |  |  |  |  |
| Genomics-informed Tx Group | 114.69 | 22.26 |  | 176.94 | 16.93 |  | 55,581.25 | 24,245.57 |  |
| Usual Care Tx Group | 116.87 | 13.30 |  | 213.64 | 22.31 |  | 17,028.08 | 6,307.351 |  |
| Difference post-sequencing (Δx̅_post_) | -2.18 | 26.28 | 0.934 | -36.70 | 27.61 | 0.184 | 38,553.17 | 24,910.00 | 0.122 |
| Incremental Effect (Δx̅) | 74.42 | 38.58 | 0.054 | -6.84 | 38.58 | 0.859 | 88,476.17 | 38,273.62 | 0.021 |

SE: standard error; WGTA: whole-genome and transcriptome analysis; Costs are reported in 2018 Canadian dollars. Significance level for within cancer type analyses: p<0.006 (=0.05/9) after Bonferroni correction.

Supplemental Table 6: Sensitivity analysis excluding patients who did not receive genomics-informed treatment because of ongoing response to usual care

| Characteristics (n=298) | Time to Treatment Discontinuation (days) | | | Time to Next Treatment (days) | | | Therapy Cost  (CAD$) | | |
| --- | --- | --- | --- | --- | --- | --- | --- | --- | --- |
|  | Mean | SE | p-value | Mean | SE | p-value | Mean | SE | p-value |
| Pre-sequencing |  |  |  |  |  |  |  |  |  |
| Genomics-informed Tx Group | 165.79 | 15.84 |  | 325.14 | 34.28 |  | 14,088.03 | 3,180.85 |  |
| Usual Care Tx Group | 243.10 | 23.00 |  | 406.82 | 40.81 |  | 27,017.10 | 6,767.66 |  |
| Difference pre-sequencing  (Δx̅_pre_= x̅_genomics,pre_- x̅_Usual,pre_) | -77.32 | 27.63 | 0.005 | -81.68 | 52.01 | 0.116 | -12,929.07 | 7,367.31 | 0.079 |
| Post-Sequencing |  |  |  |  |  |  |  |  |  |
| Genomics-informed Tx Group | 132.69 | 18.40 |  | 189.82 | 15.91 |  | 70,227.90 | 26,657.49 |  |
| Usual Care Tx Group | 123.13 | 12.82 |  | 178.76 | 14.97 |  | 11,747.67 | 2,161.70 |  |
| Difference post-sequencing  (Δx̅_post_= x̅_genomics,post_- x̅_Usual,post_) | 9.55 | 22.27 | 0.668 | 11.06 | 21.76 | 0.611 | 58,480.24 | 26,746.98 | 0.029 |
| Incremental Effect  (Δx̅=Δx̅_post_- Δx̅_pre_) | 86.87 | 33.38 | 0.009 | 92.74 | 52.10 | 0.075 | 71,409.30 | 27,934.65 | 0.011 |

Bonferroni correction was used for multiple comparisons. Significance level: p<0.006 (=0.05/9) after correction. Non-parametric bootstrapping clustered by primary tumor site estimated standard errors. Reported confidence intervals are bias-corrected. Costs are reported in 2018 Canadian dollars. SE: standard error; Tx: treatment
